# Propranolol treatment during repetitive mild traumatic brain injuries induces transcriptomic changes in the bone marrow of mice

**DOI:** 10.3389/fnins.2023.1219941

**Published:** 2023-09-12

**Authors:** Jared A. Smith, Tyler Nguyen, Brittany C. Davis, Debomoy K. Lahiri, Takashi Hato, Alexander G. Obukhov, Fletcher A. White

**Affiliations:** ^1^Department of Anesthesia, Indiana University School of Medicine, Indianapolis, IN, United States; ^2^Department of Psychiatry, Indiana University School of Medicine, Indianapolis, IN, United States; ^3^Department of Medicine, Indiana University, Indianapolis, IN, United States; ^4^Department of Anatomy, Cell Biology & Physiology, Indiana University School of Medicine, Indianapolis, IN, United States

**Keywords:** mild traumatic brain injury, sympathetic activation, immune cell, bone marrow, gene expression

## Abstract

**Introduction:**

There are 1.5 million new mild traumatic brain injuries (mTBI) annually in the US, with many of the injured experiencing long-term consequences lasting months after the injury. Although the post injury mechanisms are not well understood, current knowledge indicates peripheral immune system activation as a causal link between mTBI and long-term side effects. Through a variety of mechanisms, peripheral innate immune cells are recruited to the CNS after TBI to repair and heal the injured tissue; however, the recruitment and activation of these cells leads to further inflammation. Emerging evidence suggests sympathetic nervous system (SNS) activity plays a substantial role in the recruitment of immune cells post injury.

**Methods:**

We sought to identify the peripheral innate immune response after repeated TBIs in addition to repurposing the nonselective beta blocker propranolol as a novel mTBI therapy to limit SNS activity and mTBI pathophysiology in the mouse. Mice underwent repetitive mTBI or sham injury followed by i.p. saline or propranolol. Isolated mRNA derived from femur bone marrow of mice was assayed for changes in gene expression at one day, one week, and four weeks using Nanostring nCounter^®^ stem cell characterization panel.

**Results:**

Differential gene expression analysis for bone marrow uncovered significant changes in many genes following drug alone, mTBI alone and drug combined with mTBI.

**Discussion:**

Our data displays changes in mRNA at various timepoints, most pronounced in the mTBI propranolol group, suggesting a single dose propranolol injection as a viable future mTBI therapy in the acute setting.

## Introduction

Every year millions of individuals suffer from mild traumatic brain injuries, commonly known as concussions, that result in potentially debilitating long-term complications. Healthcare burden of treatment for concussion patients is estimated to be up to $1.35 billion annually (McGinity et al., 2018; Pavlov et al., 2019). Due to a lack of understanding of the underlying mechanisms, treatments for concussion-associated conditions, such as headache, are mostly supportive and do not effectively address the underlying pathophysiology.

Concussion can trigger a multitude of molecular processes and mechanisms which are generally ignored in favor of symptom management. These processes may eventually lead to deleterious downstream disease or injury responses. Factors influencing these responses may include tissue-specific DNA methylation, histone modification, and alterations in microRNA. These changes affect gene activation or repression and in turn determine cellular responses to a broad spectrum of environmental signals by altering protein expression.

Studies of disease genetics have successfully used the drug effects as means to identify putative genes contributing to variation in therapeutic drug responses and adverse drug reactions (Giacomini et al., 2017; Castonguay et al., 2022). Though most drugs have not been effective in treating headache symptoms associated with mTBI, some pharmacological options are available and are thought to be relatively free of serious or long term off-target effects. One such class of drugs is the beta-adrenergic receptor antagonists, so called beta-blockers. As the concussive effect of mTBI is largely thought to be associated with a hyperadrenergic state, a disrupted blood brain barrier, and high local norepinephrine levels, treatment with the non-selective beta-adrenergic receptor antagonist, such as propranolol, offers a potentially beneficial approach to blunting the cascade of post-mTBI sympathetic activation (Alali et al., 2017; Chen et al., 2017; Ding et al., 2021; Florez-Perdomo et al., 2021).

In this study, we compared the effects of propranolol on transcriptome in the bone marrow of mice subjected either to mTBI or to sham surgery. Additionally, we performed pathway enrichment analysis to identify the cellular pathways affected by propranolol alone, mTBI alone, or the combination of propranolol with head injury, focusing on those pathways that are related to fatty acid, glucose, and glutamine metabolism, stem cell epigenetic modifications, and inflammatory signaling. Our study reveals new disease-related genes and informs on the molecular and cellular basis for post-concussion sequela across time.

## Materials and methods

### Animals

All experiments were approved by the Institutional Animal Care and Use Committee (IACUC) of the Indiana University School of Medicine, which is in accordance with National Institutes of Health guidelines for the care and use of laboratory animals. 36 Wild type male C57BL/6 mice were obtained from Jackson Laboratory at 8–12 weeks old (~25–30 g) and were randomly divided into sham saline (SS), mTBI saline (TS), and mTBI propranolol (TP) and sham propranolol (SP) for downstream gene expression analyses. Mice were randomly subdivided into day 1, day 7, and day 30 timepoints with *n* = 3 for each group at each timepoint. All experiments were performed in accordance with relevant ARRIVE guidelines.

### Repeated mild traumatic brain injury procedure

**Figure 1:**
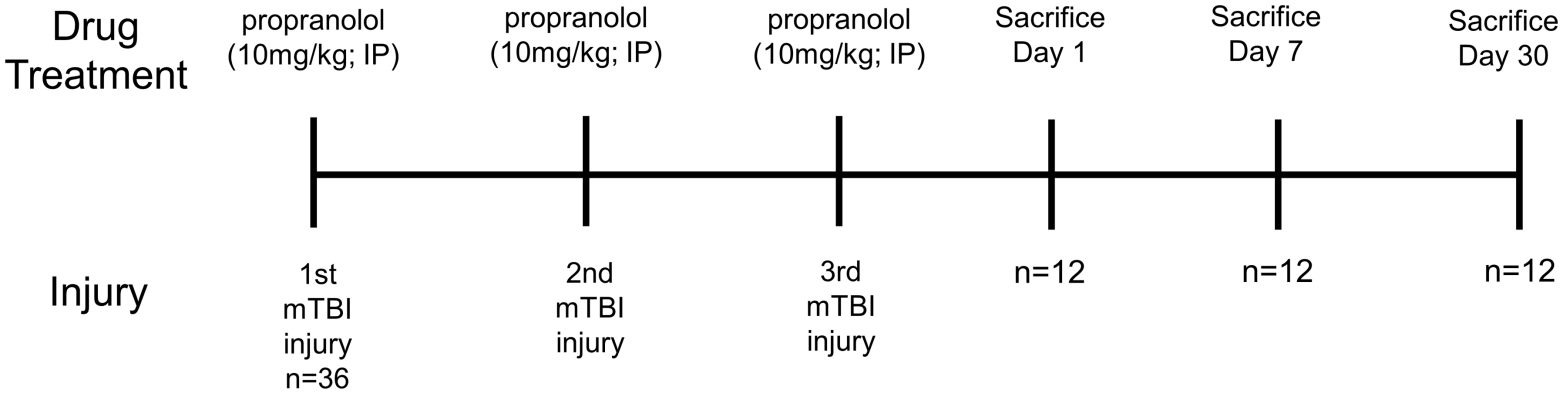
Experimental timeline.

mTBI was inflicted using a technique described recently (Nguyen et al., 2021; Smith et al., 2023). Briefly, mice were anesthetized with 2–4% isoflurane and heads stereotactically fixed with heat-pad support below the abdomen. Closed-head mTBI was produced using a control cortical impact (CCI) device after shaving, cleaning, sanitizing the area, and making a skin incision to expose the skull. After the baseline point of the device was set by lowering the tip to the exposed skull surface and the stage position was set to zero, the impactor was retracted, the impact depth was set, and the impact was made. The approximate center of the impact site was 1 mm posterior to the bregma and 0.5 mm lateral from midline over the right cortical hemisphere. The skull was struck with the impactor tip at a speed of 3 m/s to a depth of 1 mm. Sham injury animals were shaved, cleaned, sanitized, an incision was made to expose the skull, but no impact was performed on the skull. The resulting skin wound was sutured, and animal placed on heating pad until fully recovered. All animals survived the injury without any skull fracture or hemorrhage. The injury paradigm was performed once a week for three weeks for a total of 3 injuries (Figure 1).

Verification of the absence of skull fracture was assayed by changes in intraocular pressure (IOP) using ICARE TONOLAB (Vantaa, Finland) tonometer (Chen et al., 2019) and served as a surrogate measure for internal brain pressure. The IOP measurements were done immediately before and after the injury for up to 30 min (data not shown). Predictably, if the skull calvarium was fractured by the CCI device, an absence of IOP would be present. Alternatively, if there were no changes in IOP, injury to the skull bone was deemed to be insufficient and the animal excluded from the study. No animals were subsequently removed from this study.

### Propranolol administration

Propranolol chloride (Tocris Bioscience, Bristol, United Kingdom) was dissolved in saline (3 mg/mL). Based on the treatment group designation, the drug (10 mg/kg) or saline vehicle was injected intraperitoneally into each mouse immediately following mTBI or sham surgery as previously stated by other studies (Haffner-Luntzer et al., 2019; Loftus et al., 2019). Each mouse was subjected to a total of three of either propranolol or saline injections.

### Bone marrow tissue processing

We followed previously described bone marrow protocols to extract bone marrow tissue from the femurs of mice (Marim et al., 2010). Briefly, scissors were used to cut the femur from the pelvis and knee joint. The femur was then placed into an ice cold PBMC culture medium composed of complete RPMI medium (88% RPMI 1640; ThermoFisher), 10% Fetal Bovine Serum (Sigma-Aldrich), 1% Penicillin-Streptomycin (Sigma-Aldrich), and 1% Glutamax (ThermoFisher). Next, a 23G needle attached to a 3 mL syringe filled with ice cold complete RPMI was inserted into the medullary cavity of the femur and the bone marrow was flushed out into a 15 mL tube. The procedure was repeated 2–3 times until the femur diaphysis was white. The resultant tissue was centrifuged at 400xg for 10 min at 4°C, and the supernatant was discarded. Remaining tissue was then resuspended in RBC lysis buffer for 10 min at room temperature, recentrifuged, and resuspended in calcium free PBS. The suspension was again centrifuged at 400xg for 10 min at room temperature, resuspended in complete RPMI medium, and then added to the equal volume of FBS supplemented with 10% DMSO for freezing. The frozen bone marrow was stored in liquid nitrogen at −196°C until future analyses.

### NanoString nCounter gene expression panels

mRNA was isolated from each bone marrow sample using the TRIzol reagent (Invitrogen) as described in the manufacturer’s protocol. RNA samples were evaluated by nCounter gene expression analysis technology (NanoString Technologies) and quantified via nCounter Digital Analyzer (NanoString Technologies). The expression of 770 genes (including 14 internal reference genes) was determined using the nCounter Stem Cell Characterization Panel™ (mouse, NanoString, XT-CSO-MSCC-12). To minimize variability among arrays, densitometry values between arrays were normalized using Robust Multichip Average function and further transformed to a log2 scale. Gene expression levels in each sample were normalized against the geometric mean of six housekeeping genes, specifically Cltc, Gapdh, Tpia, Tbp, Pgk1, and Tubb5. A cutoff was introduced at the value of the highest negative control present on the chip. 100 ng of total mRNA was used as input and sample hybridization was performed according to the manufacturer’s instructions. Raw data processing, quality control (QC), and normalization were performed using the nSolver™ 4.0 analysis software. QC and normalization were performed with an imaging QC of >75% field of view registration, binding density QC within 0.1–2.25 range, positive control linearity QC of R2 above 0.95, and positive control limit of detection set as 0.5 fM positive control above 2 standard deviations above the mean of the negative controls. Normalization to housekeeping genes, of which genes below 100 were excluded, and pathway scoring, gene set analysis, differential expression analysis, and cell type profiling were completed using the Advanced Analysis software plugin (version 2.0.115). For each experiment, the fold changes were calculated comparing to their appropriate sham-injured saline treated control at each timepoint. For pathway scoring and differential expression analysis, a *value of p* of ≤0.05 was applied as cutoffs. For all NanoString analyses at 1 day, 1 week, and 1 month after injury, gene expression measurements for each group were normalized to the sham saline (*n* = 3) baseline of each timepoint.

### Differential expression

**Figure 2:**
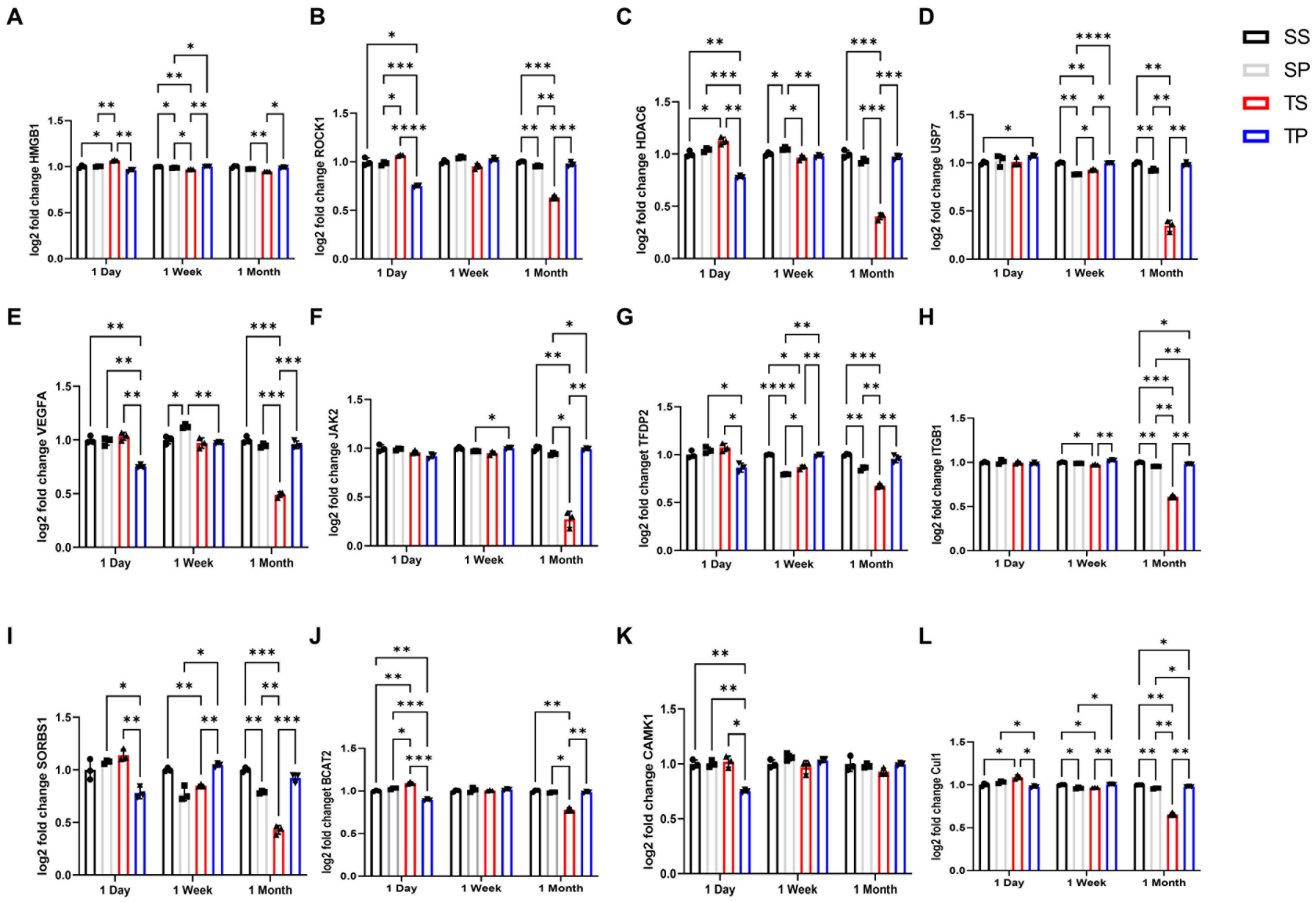
Effect of injury alone or in combination with propranolol on genes in bone marrow. Fold change in mRNA expression between sham injury plus saline tissue (black bar), sham injury plus propranolol (light gray bar), rmTBI plus saline (dark gray) and rmTBI plus propranolol (hatched bar) at one day, seven days and one month following injury. **(A)** HMGB1, **(B)** ROCK1, **(C)** HDAC6, **(D)** USP7, **(E)** VEGFA, **(F)** JAK2, **(G)** TFDP2, **(H)** ITGB1, **(I)** SORBS1, **(J)** BCAT2, **(K)** CAMK1, AND **(L)** CUL1 values represent mean ± SEM of one experiment in triplicate. Data is expressed as log2 count statistical analysis by 2-way ANOVA followed by Sidak’s multiple comparison test (*p* < 0.05) *, (*p* < 0.01) **, (*p* < 0.001) ***, (*p* < 0.0001) ****.

Differentially expressed (DE) genes were calculated using fold change by comparing transcript levels between the combined samples within a group of SP, TS, or TP bone marrow and sham saline bone marrow baseline. The results of these multiple *t* test analyses are summarized in volcano plots, (Tables 1–3), and in Supplementary Data Sheets S1–S6. Data was graphed −log10(value of *p*) vs. fold change with Bonferroni adjusted *p* value dotted lines shown to depict varying levels of significance at *p* < 0.50, *p* < 0.10, *p* < 0.05, and *p* < 0.01. 12 of the most significantly changed genes across the experimental groups and timepoints were chosen for further characterization (Figure 2). These data were generated by NanoString advanced analysis and selected due to high differential expression. Log2 fold changes for these mRNAs were further assessed across the experimental timepoints to determine group dependent changes for these transcripts.

### Pathway scores

Panel derived transcripts from each sample were grouped into pathways by Nanostring Advanced Analysis software and Scores are displayed on the same scale via a *Z*-transformation. Mean *z* score for each group was compared at each timepoint to the sham saline baseline control to understand how pathway scores cluster together and which samples exhibit similar pathway score profiles at each timepoint.

### Statistics

NanoString data normalized to housekeeping gene expression were initially analyzed using the *t*-test to identify the top 100 significant genes. The Bonferroni correction was applied to derive the adjusted *p* values with a ≤0.05 with a cut off adjusted *p* value of ≤0.05. Sham saline data were utilized as baseline for each timepoint. Gene expression changes were reported as log2 fold change. Expression change data were compared using two-way ANOVA (treatment vs. time) test followed by the Šidák multiple comparisons *post hoc* test (GraphPad Prism 9, La Jolla, California). Pathway score significance was calculated using unpaired *t*-test, with *p* < 0.05 being considered significant, and mean values were calculated by averaging pathway Z scores of individual samples within a group at one timepoint (*n* = 3/group). All data were presented as mean ± S.E.M. The sample size was *n* = 3 for each tested group.

## Results

### A high-resolution transcriptional profile of repeated mTBI in mice subjected to propranolol or saline treatment

Figure 1 shows the timeline of the experiments. Three mice per treatment group were used for each post-injury/drug treatment time point in this study (Figure 1). This gave a detailed coverage of acute, subacute and chronic responses of bone marrow tissue to rmTBI in the absence or presence of propranolol treatment.

### Effect of propranolol combined with repeated mTBIs at 1-day post-injury

**Figure 3:**
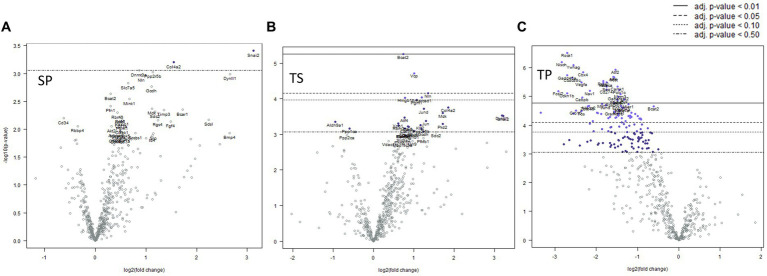
Volcano plots showing differential gene expression in bone marrow caused by the following treatments at one day: **(A)** sham injury plus propranolol (SP), **(B)** rmTBI plus saline (TS), and **(C)** rmTBI plus propranolol (SP). Sham plus saline is utilized as the baseline comparator value. Data is expressed log2 fold change vs. *p* value and graphed with Bonferroni adjusted *p* value lines to denote levels of significance; *n* = 3.

Mice sacrificed on the day 1 following completion of the 3x injury/drug treatment paradigm showed no significant changes of gene expression in SP treatment group (Figure 3A), whereas the TS treatment group exhibited increased gene expression in branched chain amino acid transaminase (*Bcat2*), valosin-containing protein (*Vcp*) and neurolysin (*Nln*) (Figure 3B; Supplementary Data Sheet S1). The TP treatment group exhibited the greatest change at day 1 as there were 66 genes which displayed decreased expression including *Bcat2* (Figure 3C; Supplemetary Data Sheet S2).

Complex biological processes, such as those present in bone marrow tissue involve the coordinated regulation of multiple intracellular signaling pathways controlling gene expression. The signaling pathway changes found in the SP treatment group were limited to an increase in Hedgehog signaling (Table 1). In contrast, the decreases in gene expression observed in both TS and TP bone marrow tissue were accompanied by decreases in signaling pathway scores on the day 1 time point following injury paradigm for all assayed pathways (Table 1, *p* < 0.05, *t* test).

**Table 1:** Pathway scores for each timepoint of the experiment.

1 Day											
Bone marrow pathway	SS mean	SD	SP mean	SD	SP *p* value	TS mean	SD	TS *p* value	TP mean	SD	TP *p* value
Amino acid metabolism	0.494378	0.576169	1.161931	0.506661	0.206291	1.87804	0.446902	0.030309	−3.53435	0.710825	0.001588
AP-1 signaling	1.118391	0.208951	0.525142	0.951839	0.351167	2.446945	0.73121	0.038938	−4.09048	0.085971	2.35E-06
Apoptosis	0.899437	0.646981	1.296304	0.886683	0.565112	2.874318	0.546626	0.015623	−5.07006	0.26861	0.000123
Autophagy	0.606031	0.506829	1.091482	0.472516	0.291713	2.95496	0.455623	0.003955	−4.65247	0.380292	0.000136
Cell cycle	0.717299	0.595177	0.895902	0.697012	0.752696	2.484946	0.783314	0.035797	−4.09815	0.207988	0.000189
Cytoskeletal reorganization	0.921307	0.76362	1.80195	0.612716	0.194242	3.339124	0.455114	0.009234	−6.06238	0.993948	0.000645
Ectodermal lineage	0.823892	0.446727	1.281209	0.909058	0.477907	2.8757	0.380806	0.003757	−4.9808	0.661064	0.000228
Endodermal lineage	0.521953	0.424573	0.940205	0.990491	0.538269	2.204332	0.561316	0.014373	−3.66649	0.757199	0.001121
Epigenetic modification	0.804199	0.344388	1.635704	1.081473	0.273282	3.353635	0.500473	0.001903	−5.79354	0.800385	0.000195
Fatty acid metabolism	0.58208	0.178463	0.688447	0.495	0.743901	2.057177	0.531604	0.010369	−3.3277	0.560371	0.000325
Glucose metabolism	0.746466	0.658223	1.038541	0.74673	0.638047	3.037124	0.464053	0.007893	−4.82213	0.459971	0.000275
Glutamine metabolism	0.118851	0.278374	0.472951	0.188788	0.142308	0.407707	0.230926	0.238776	−0.99951	0.051581	0.002388
Hedgehog signaling	0.154187	0.246596	0.644338	0.135442	0.039258	1.479775	0.123389	0.001137	−2.2783	0.227025	0.000231
Hippo signaling	0.261287	0.512088	1.010149	0.750255	0.226498	2.071795	0.540602	0.013572	−3.34323	0.363268	0.000574
HOX gene activation	0.453373	0.135844	0.559632	0.435505	0.707265	1.377314	0.509094	0.038508	−2.39032	0.248877	6.45E-05
Hypoxia response	0.849981	0.326266	0.829158	0.669115	0.963681	1.836648	0.423078	0.032942	−3.51579	0.534153	0.000269
Integrin signaling	0.548606	0.283324	1.279533	0.369302	0.052994	1.697781	0.40777	0.016015	−3.52592	0.99147	0.002385
JAK–STAT signaling	1.073126	0.646997	0.622989	0.785546	0.486322	2.240607	0.568061	0.078633	−3.93672	0.536962	0.000497
MAPK signaling	1.194454	0.547392	1.161904	1.14088	0.966598	3.408976	0.66216	0.011121	−5.76533	0.742397	0.000198
Mesodermal lineage	0.473005	0.558507	0.908942	1.093349	0.571816	2.690598	0.494641	0.006752	−4.07255	0.779949	0.001201
MET & EMT signaling	1.012264	1.291534	1.829555	1.42926	0.503185	3.651212	0.282994	0.025888	−6.49303	1.381741	0.002348
mTOR signaling	0.617896	0.585413	0.957085	0.774036	0.577606	2.465222	0.533687	0.015615	−4.0402	0.234019	0.000215
Na.QZ.ve State	0.380254	0.037558	0.515349	0.746943	0.770013	1.586532	0.326523	0.003139	−2.48213	0.208932	1.99E-05
Notch signaling	0.249961	0.310971	0.792892	0.644441	0.259069	1.772447	0.459577	0.008956	−2.8153	0.360961	0.000369
Oxidative stress response	1.113736	0.755951	0.998199	0.980418	0.879424	3.289633	0.641217	0.019072	−5.40157	0.616116	0.000319
Partially reprogrammed	0.327434	0.204131	0.114537	0.353851	0.417744	1.056643	0.507062	0.081973	−1.49861	0.263964	0.000691
PI3K-AKT signaling	0.993399	0.934733	1.726785	1.045653	0.416323	3.586572	0.449191	0.01234	−6.30676	1.167141	0.001072
Pluripotency markers and regulators	0.904365	0.630075	1.209445	1.004668	0.678953	2.958129	0.677282	0.018374	−5.07194	0.680691	0.000367
Primed State	−0.01819	0.161025	0.451902	0.513536	0.204852	0.840093	0.162143	0.002881	−1.2738	0.304136	0.003208
RhoROCK signaling	0.426213	0.325343	0.344664	0.313389	0.770146	1.262951	0.33329	0.035815	−2.03383	0.269287	0.000543
Senescence and quiescence	1.17159	0.599645	1.14015	0.898997	0.962226	3.100459	0.741928	0.024848	−5.4122	0.385639	8.93E-05
TGF-beta signaling	0.337498	0.405265	1.10312	0.468421	0.098984	2.36848	0.357188	0.00287	−3.8091	0.327473	0.000161

Means for each group at one day after injury. Significant pathway scores were compared using *t*-test vs. sham saline as comparison at each timepoint (*p* < 0.05).

### Effect of propranolol combined with repeated mTBI at 7-days post-injury

**Figure 4:**
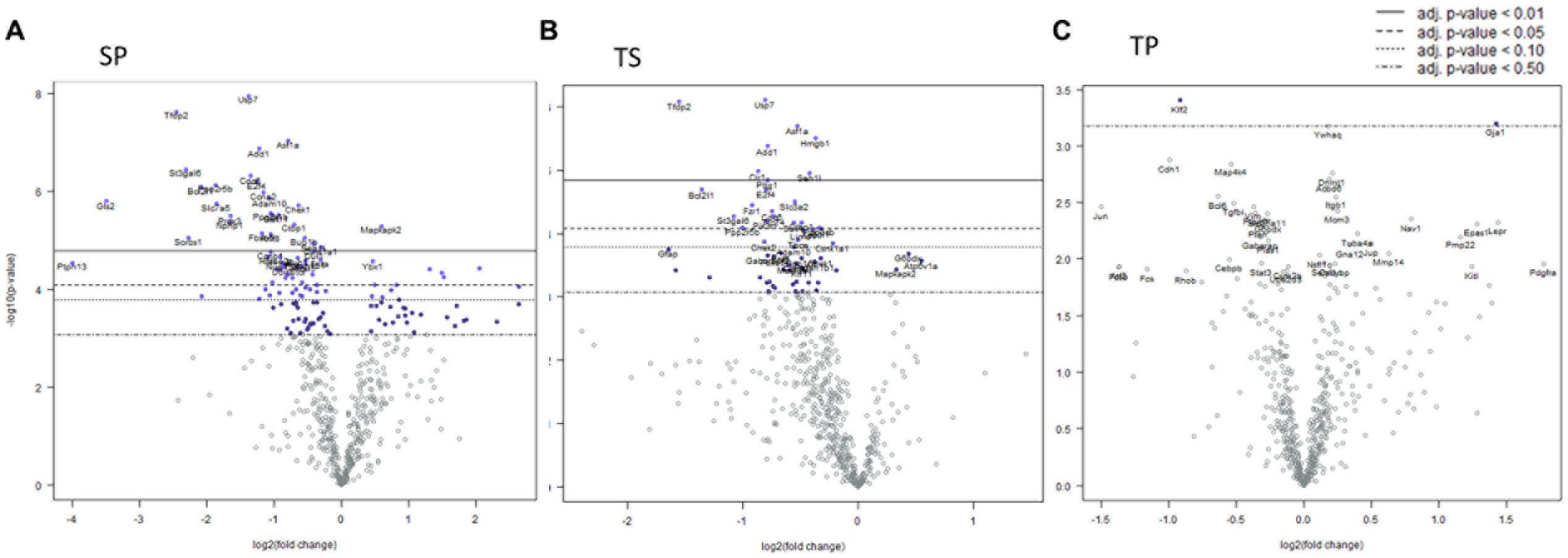
Volcano plots of mRNA fold changes in bone marrow one week post injury. **(A)** Sham propranolol (SP), **(B)** rmTBI saline (TS), and **(C)** rmTBI propranolol (TP). Sham plus saline is utilized as the baseline comparator value. Data is expressed log2 fold change vs. *p* value and graphed with Bonferroni adjusted *p* value lines to denote levels of significance; *n* = 3.

Seven days post cessation of the 3x injury/drug treatment paradigm, the SP group exhibited 57 genes which were significantly different when compared with the SS group (Figure 4A, *p* < 0.05, *t* test). Of this group, 48 genes exhibited decreased expression and 9 genes showed increase expression (Figure 4A; Supplementary Data Sheet S3). A significant decreased expression for 21 genes in the TS group were evident (Figure 4B; Supplemetary Data Sheet S4). The SP and TS groups at 7 days exhibited similar decreases in *Usp7, tfdp2, add1, pp2r5b1, cir1,* and *cul1*. Unlike the day 1 post injury paradigm, TP bone marrow failed to exhibit either increases or decreases in gene expression at 7 days (Figure 4C).

Signaling pathway changes in SP bone marrow tissue exhibited the greatest number of pathway changes (26 of 32) at 7 days post-injury (Table 2). The SP bone marrow tissue displayed decreases in amino acid metabolism, apoptosis, autophagy, cell cycle, cytoskeletal reorganization, epigenetic modifications, glutamine metabolism, hedgehog signaling, notch signaling, senescence and quiescence, and TGFβ signaling (Table 2, *p* < 0.05, *t* test). Increases in signaling pathway were also apparent. Significant changes were found in AP1 signaling, ectodermal lineages, endodermal lineage, fatty acid metabolism, glucose metabolism, hippo signaling, hypoxia response, MAPK signaling, mesodermal lineages, mTOR signaling, Na.QZ.ve state, oxidative stress, partially reprogrammed, pluripotency markers, and RhoROCK signaling (Table 2).

**Table 2:** Pathway scores for each timepoint of the experiment.

7 Day											
Bone marrow pathway	SS mean	SD	SP mean	SD	SP *p* value	TS mean	SD	TS *p* value	TP mean	SD	TP *p* value
Amino acid metabolism	0.777847	0.235572	−2.09817	0.401278	0.000431	0.040574	0.27054	0.023591	1.279749	0.31293	0.090671
AP-1 signaling	−0.76533	1.003159	2.945952	0.09002	0.003093	0.050138	1.024084	0.380275	−2.23076	0.330611	0.074109
Apoptosis	1.013183	0.663077	−2.90566	0.064452	0.000523	0.010931	0.754977	0.159124	1.881543	0.324798	0.111319
Autophagy	1.122116	0.042994	−1.44157	0.2353	4.96E-05	−1.30992	0.389611	0.000425	1.629375	0.358135	0.071534
Cell cycle	1.392324	0.28484	−2.3985	0.130338	3.06E-05	−0.68667	0.406974	0.001923	1.692848	0.243309	0.237023
Cytoskeletal reorganization	0.484899	1.109402	−0.08401	0.818565	0.514279	−2.63552	0.870473	0.018574	2.234638	1.2542	0.144559
Ectodermal lineage	−0.42097	0.718295	2.193992	1.020926	0.022192	−1.98315	1.265921	0.136559	0.21013	0.325845	0.238047
Endodermal lineage	−0.58425	0.903672	2.009206	1.031494	0.030627	−1.72727	1.196321	0.25716	0.302318	0.521949	0.215133
Epigenetic modification	1.109402	0.503615	−2.60799	0.204901	0.000291	−0.27384	0.431352	0.022494	1.772425	0.107475	0.089603
Fatty acid metabolism	−0.67818	0.515186	1.655226	0.320886	0.002642	−0.30002	0.603936	0.455673	−0.67703	0.149667	0.997205
Glucose metabolism	−1.00589	0.59229	2.674955	0.336017	0.000725	−0.60884	0.832889	0.537886	−1.06023	0.245571	0.890404
Glutamine metabolism	0.29675	0.138246	−0.91472	0.428532	0.009589	−0.14574	0.117133	0.013373	0.763705	0.129872	0.013012
Hedgehog signaling	0.302611	0.186796	−0.40515	0.311918	0.027998	−0.83156	0.155495	0.001273	0.934097	0.257135	0.026259
Hippo signaling	−0.68037	0.262753	2.032259	0.246068	0.000199	−0.15969	0.353395	0.109951	−1.1922	0.184599	0.050813
HOX gene activation	0.038714	0.304698	0.791653	0.426033	0.067496	−1.03097	0.548759	0.041896	0.200602	0.143505	0.451927
Hypoxia response	−0.11669	0.500465	1.372066	0.498139	0.021737	−1.5647	1.060807	0.099283	0.309316	0.34804	0.292749
Integrin signaling	0.233658	0.876046	0.439658	0.64986	0.759986	−1.88093	0.942639	0.046578	1.20761	1.156816	0.309654
JAK–STAT signaling	−0.06056	0.377	1.053135	1.019065	0.150505	−2.00038	1.074446	0.04194	1.007815	0.546784	0.049502
MAPK signaling	−0.34309	0.840891	2.375641	0.771146	0.014526	−2.09859	1.409828	0.137626	0.06604	0.33374	0.477243
Mesodermal lineage	−0.91072	0.991745	2.101814	0.906547	0.01779	−1.32212	0.767914	0.600343	0.131029	0.692986	0.210131
MET & EMT signaling	−0.63466	1.565773	2.395771	1.290558	0.060888	−3.44798	1.854917	0.115139	1.686873	1.418041	0.129718
mTOR signaling	−0.91529	0.385349	1.85116	0.329108	0.000698	−0.05961	0.554931	0.093306	−0.87626	0.328943	0.900302
Na.QZ.ve State	−0.36971	0.424278	1.511186	0.651446	0.013801	−0.35828	0.591706	0.979596	−0.7832	0.124481	0.180609
Notch signaling	1.193989	0.281779	−2.12449	0.051414	3.64E-05	−0.2633	0.275801	0.003058	1.193808	0.146267	0.999259
Oxidative stress response	−0.8347	0.957204	3.047295	0.392515	0.002891	−0.96201	1.449814	0.90512	−1.25058	0.466729	0.535871
Partially reprogrammed	−0.29964	0.243851	0.603048	0.39628	0.028299	0.340977	0.174147	0.020782	−0.64438	0.270282	0.176293
PI3K-AKT signaling	−0.21345	1.19366	1.625884	1.369481	0.154355	−2.94195	1.75435	0.089893	1.529523	1.194378	0.148325
Pluripotency markers and regulators	−0.37208	0.951836	2.63134	1.131905	0.024506	−1.8482	1.063695	0.147746	−0.41107	0.501586	0.952962
Primed State	0.343002	0.079891	−0.46551	0.547294	0.064533	−0.46127	0.213424	0.003626	0.58378	0.187725	0.110423
RhoROCK signaling	−0.18702	0.23935	1.252554	0.291077	0.002705	−0.60005	0.691921	0.383847	−0.46548	0.079081	0.128254
Senescence and quiescence	1.306359	0.535407	−2.99902	0.033089	0.000155	−0.41068	0.526519	0.016671	2.10334	0.195639	0.072634
TGF-beta signaling	0.994003	0.483484	−2.37853	0.256455	0.000436	0.213306	0.509846	0.126618	1.171225	0.118714	0.57088

Shown are the means for each group at one week after injury. Significant pathway scores were compared using the *t*-test with sham saline as comparison at each timepoint (*p* < 0.05).

TS bone marrow tissue exhibited numerous changes in 15 of 32 signaling pathways (Table 2). These changes were largely reductions in signaling pathways such as, autophagy, cell cycle, cytoskeletal reorganization, epigenetic modifications, glutamine metabolism, hedgehog signaling, HOX gene activation, integrin signaling, JAK–STAT signaling, notch signaling, primed state activation, and senescence and quiescence. Significant increases were found only in three signaling pathways such as amino acid metabolism, AP-1 signaling, and partially reprogramed cells (Table 2, *p* < 0.05).

Despite a lack of gene expression changes in the TP animal group, we observed significant increases in signaling pathway scores for glutamine metabolism, hedgehog signaling, and JAK–STAT signaling. Increases in signaling pathways were limited to only glutamine metabolism in TP bone marrow when compared to TS bone marrow (Table 2).

### Effect of propranolol combined with repeated mTBI at 30-days post-injury

**Figure 5:**
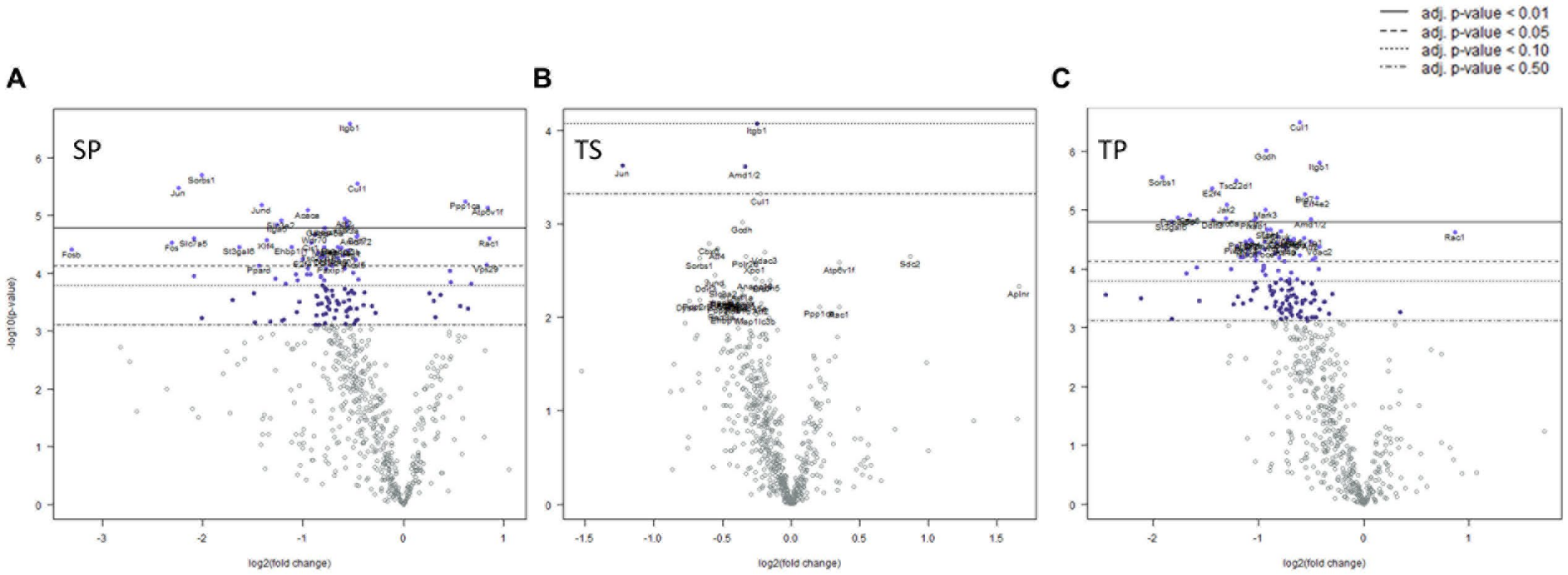
Volcano plots of mRNA fold changes in one month post injury bone marrow **(A)** Sham propranolol (SP), **(B)** rmTBI saline (TS), and **(C)** rmTBI propranolol (TP). Sham plus saline is utilized as baseline comparator value. Data is expressed log2 fold change vs. *p* value and graphed with Bonferroni adjusted *p* value lines to denote levels of significance; *n* = 3.

We observed significant differential expression of 40 genes in the SP group and 52 genes in the TP group but no changes in gene expression in the TS group at 30-days (Figure 5B) after completion of the injury/drug treatment paradigm. Among the genes which exhibited decreased expression in both treatment conditions of SP and TP were *Cul1*, *Itgb1*, *Sorbs1*, *Tsc22d1*, *E2f4, Brd7, Mark3, Cir1*, and *Amd1/2* (Figures 5A,C; Supplementary Data Sheets S5, S6). This differential gene expression profiling clearly demonstrates the long-lasting impact of propranolol on bone marrow tissue in both SP and TP animal groups. None of the genes associated with TS met the established criterion for significant change in expression levels.

Characterization of signaling path way changes following the injury/drug treatment paradigm demonstrated significant signaling pathway changes present in SP, TS, and TP bone marrow tissues 30 days after injury. The SP bone marrow tissue exhibited significantly decreased signaling in 25/32 pathways when compared to the SS bone marrow tissue. The pathway score changes for the various groups are summarized in Table 3. TS bone marrow had increased signaling in amino acid metabolism, apoptosis, cell cycle signaling, cytoskeletal reorganization, glucose metabolism, hedgehog signaling, and oxidative stress pathways. Additional signaling changes appeared in the TP bone marrow tissue 30 days following injury, as shown by significant decreases in 24/32 pathways compared to baseline SS tissue. Here, we again observed a high crossover of decreased signaling changes common between SP and TP bone marrow tissues. Signaling pathway changes that differed between SP and TP were limited to an increase in AP-1 signaling. Compared to TS bone marrow tissue, rmTBI bone marrow from TP mice exhibit significant decreases in 24/32 signaling pathways.

**Table 3:** Pathway scores for each timepoint of the experiment.

30 Days											
Bone marrow pathway	SS mean	SD	SP mean	SD	SP *p* value	TS mean	SD	TS *p* value	TP mean	SD	TP *p* value
Amino Acid Metabolism	1.596149	0.331351	−1.01136	0.357345	0.000754	0.654777	0.318588	0.023859	−1.23957	0.682011	0.002927
AP-1 Signaling	1.838433	0.841286	−2.21866	0.238018	0.001301	0.247064	0.787472	0.075008	0.133159	0.636603	0.048829
Apoptosis	2.257475	0.372731	−1.73673	0.415936	0.000244	0.770826	0.535297	0.016852	−1.29157	0.152096	0.000107
Autophagy	1.948828	0.4514	−0.96037	0.310758	0.000777	0.843947	0.571971	0.058405	−1.8324	0.494182	0.000611
Cell Cycle	2.183239	0.531967	−0.90239	0.231093	0.000771	0.797104	0.644247	0.045304	−2.07795	1.103416	0.003823
Cytoskeletal Reorganization	2.604489	0.63306	−1.67867	0.32538	0.000479	1.026248	0.403735	0.021951	−1.95207	0.288215	0.000344
Ectodermal Lineage	1.665119	0.729141	−1.45697	0.433878	0.003109	0.992947	0.548636	0.271051	−1.20109	0.586057	0.006059
Endodermal Lineage	0.969628	1.630993	−1.5455	0.848098	0.076843	0.878826	0.584352	0.932034	−0.30296	0.623684	0.275425
Epigenetic Modification	2.347294	0.923439	−1.52902	0.49149	0.003029	0.856993	0.868983	0.11149	−1.67527	0.182714	0.001777
Fatty Acid Metabolism	0.338992	0.281348	0.674974	0.172115	0.152428	0.383011	0.557304	0.908687	−1.39698	0.903633	0.033631
Glucose Metabolism	2.031268	0.41104	−1.77112	0.192797	0.000131	0.8726	0.388939	0.023875	−1.13275	0.148623	0.000233
Glutamine Metabolism	0.577038	0.34158	−0.48053	0.123864	0.007275	0.123971	0.096001	0.091455	−0.22048	0.151983	0.020932
Hedgehog Signaling	1.044056	0.176432	−0.77596	0.26494	0.000584	0.395355	0.347068	0.044748	−0.66345	0.064346	9.50E-05
Hippo Signaling	1.283835	0.533464	−0.99798	0.604959	0.008044	0.810572	0.049502	0.200755	−1.09643	0.615543	0.007173
HOX Gene Activation	0.924549	0.534789	−0.54455	0.192473	0.011016	0.31425	0.477297	0.214315	−0.69425	0.13194	0.00703
Hypoxia Response	0.913719	0.794009	−1.0293	0.272098	0.016002	0.302584	0.629282	0.355098	−0.187	0.29607	0.087665
Integrin Signaling	1.049514	1.003146	−1.09786	0.920186	0.05232	0.265482	0.470328	0.287543	−0.21714	0.65885	0.141553
JAK–STAT Signaling	1.574711	0.613816	−0.86	0.393544	0.00444	0.963456	0.452668	0.237399	−1.67816	0.577024	0.0026
MAPK Signaling	1.955863	1.165885	−1.69969	0.626852	0.008755	1.353141	0.864903	0.511831	−1.60931	0.563746	0.008851
Mesodermal Lineage	0.392474	1.722343	−1.72391	0.530552	0.111703	0.738549	1.021538	0.779593	0.592889	1.011425	0.870468
MET & EMT Signaling	1.448016	2.985378	−2.78316	1.743242	0.101355	1.564443	1.781011	0.956523	−0.2293	1.502673	0.433766
mTOR Signaling	1.705623	0.489601	−1.11822	0.221929	0.000809	0.67234	0.44434	0.053711	−1.25975	0.279196	0.000804
Na.QZ.ve State	0.599439	0.387984	0.151266	0.142826	0.133656	0.415516	0.136944	0.481996	−1.16622	0.110323	0.001623
Notch Signaling	1.68873	0.372891	−0.91215	0.250267	0.000555	0.967088	0.484385	0.11035	−1.74366	0.726639	0.001893
Oxidative Stress Response	2.214176	0.659433	−2.37226	0.477547	0.000618	0.711706	0.607222	0.043983	−0.55362	0.421417	0.003598
Partially Reprogrammed	0.770609	0.499989	−0.71484	0.253126	0.010099	0.393061	0.218583	0.296916	−0.44884	0.477803	0.037877
PI3K-AKT Signaling	2.323036	1.607488	−2.40129	1.117558	0.013923	1.307082	0.864196	0.389557	−1.22883	0.995625	0.03127
Pluripotency Markers & Regulators	1.334853	1.430248	−1.60934	0.681201	0.032311	1.105139	0.391035	0.801709	−0.83066	0.907793	0.091206
Primed State	0.409399	0.242974	−0.50823	0.207654	0.007637	0.168554	0.140499	0.211401	−0.06972	0.253285	0.077292
RhoROCK Signaling	0.812325	0.508028	−0.72042	0.342963	0.012339	0.094092	0.624414	0.19714	−0.186	0.628982	0.099238
Senescence and quiescence	2.313808	0.727065	−1.01943	0.344701	0.001998	0.966863	0.699383	0.081804	−2.26124	0.796605	0.001827
TGF-beta Signaling	1.662541	0.468425	−1.18247	0.232928	0.000708	0.864347	0.268569	0.06261	−1.34442	0.389371	0.001027

Shown are the means for each group at one month after injury. Significant pathway scores were compared using the *t*-test with sham saline as comparison at each timepoint (*p* < 0.05).

### Long term bone marrow tissue mRNA changes affect expression of several genes important for numerous signaling pathways

Expression of numerous genes were affected by the drug/injury paradigm across the three timepoints (Figure 2; Table 3). SP group changes at day 1 were most evident by decreased gene expression of *hmgb1, rock1, hdac6, vegfa, tfdp2, sorbs1, bcat2, camk1, and cul1* when compared to SS treated animal mRNA transcripts. The only gene expression which increased at day 1 was *usp7* (*p* < 0.05, two-way ANOVA, Šidák *post hoc*). This change was restricted to a comparison between sham saline (SS) bone marrow and not to rmTBI (TS) bone marrow.

Seven days after injury TP mRNA transcripts were increased for *hmgb1, usp7, tfdp2, itgb1, sorbs1,* and *cul1* when compared to TS derived bone marrow mRNA expression levels by two-way ANOVA with Šidák *post hoc* test. *Jak2* was also increased in TP bone marrow when compared to SP bone marrow tissue. Two gene transcripts, *hdac6* and *vegfa*, were decreased in TP bone marrow but only when compared to SP bone marrow. Interestingly, TP genes did not differ from SS derived gene transcripts for the genes we investigated at this timepoint.

Two-way ANOVA testing for the 30-day timepoint displayed genetic changes for several gene transcripts derived from TP animals. We observed increased *hmgb1, rock1, hdac6, usp7, vegfa, jak2, tfdp2, itgb1, sorbs1, bcat2,* and *cul1* compared to the TS-derived bone marrow tissue (Supplementary Data Sheets S5, S6). It is worth noting that some gene expression increases in the TP group were considerably less when the data were compared to SS-derived bone marrow tissue, specifically for *itgb1* and *cul1* genes. It should be noted however that for the ANOVA analyses of these 12 genes, we detected a significant interaction between our two variables of treatment and time.

### Long-term impact of propranolol treatment after TBI

**Figure 6:**
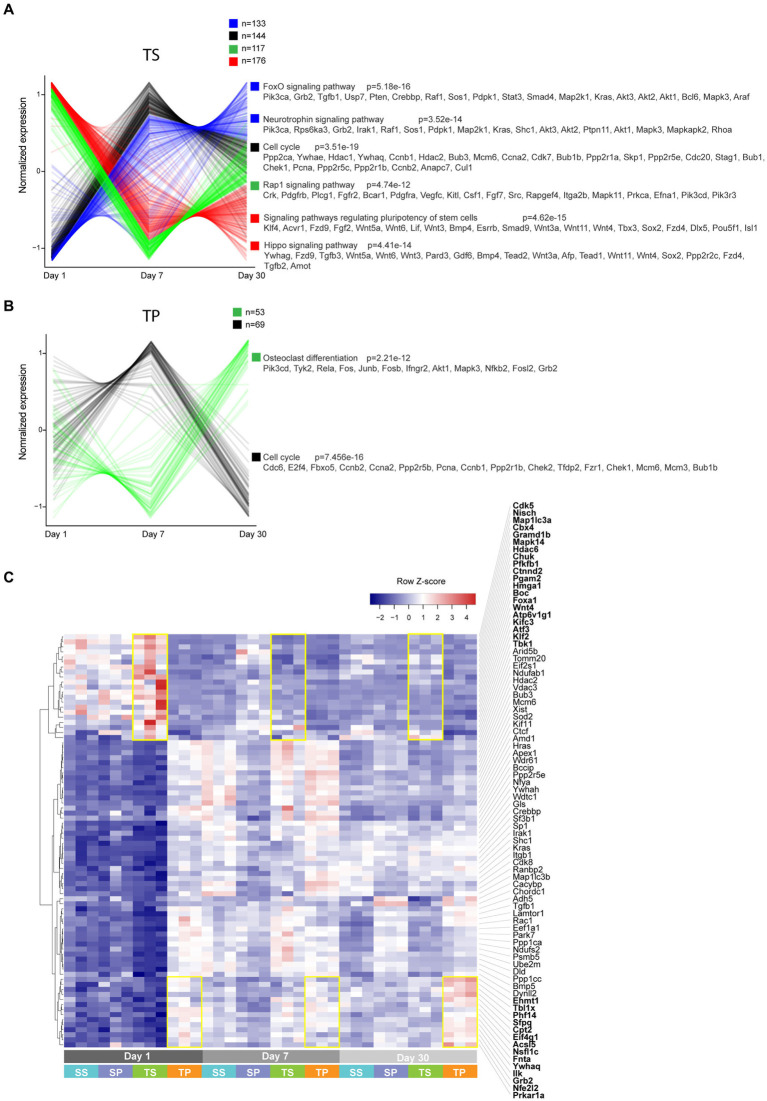
**(A,B)** Unsupervised clustering analysis performed on genes that exhibited time-dependent expression changes. Clusters are color-coded, and corresponding enriched KEGG pathways are shown. **(C)** Heatmap of select genes based on a model matrix accounting for a time effect. Yellow rectangles highlight subsets of genes that are increased in TS vs. TP (top) and TP vs. TS (bottom) across timeline.

We next performed pathway enrichment analysis on genes that exhibited timeline-specific changes (Figures 6A,B). More than 500 genes showed temporal expression changes in TS bone marrow tissue. In contrast, only 122 genes had time-dependent changes in TP, suggesting that propranolol largely blunted gene expression changes. In general, genes involved in cell proliferation, differentiation, and stem cell renewal were enriched in TS bone marrow tissue (FoxO, neurotrophin, cell cycle, pluriopotency, and Hippo signaling pathways). Similar classes of genes were enriched in TP (90 out of 122 genes are shared with TS). Finally, using a linear regression model, we searched for genes that are differentially expressed between TS and TP independently of the TBI timeline. This analysis revealed only modest enrichment of genes that still showed some degrees of time-dependent effects (Figure 6C; highlighted in yellow rectangles as an example).

## Discussion

Deciphering transcriptomic changes elicited by rmTBI is important for understanding the cellular and molecular mechanisms underlying post-concussion complications. Beta-blockers are widely used in TBI patients, and it is important to determine how such treatment modulates TBI-associated changes in gene expression and whether it affects any cellular signaling pathways. In this study we investigated the effects of propranolol treatment on bone marrow tissue derived from mice subjected to repetitive mild trauma to the calvarium. TBI is known to cause systemic neuro-inflammation, whereas bone marrow generally provides a framework of microenvironmental domains or niches that support the hematopoietic and immune system and is critical for body responses to infections and injuries. Our data revealed that propranolol treatment variably modulated TBI-induced changes in gene expression and signaling pathways within bone marrow tissue.

Bone marrow dysfunction is common in severely injured trauma patients as the tissue is known to be influenced by factors including elevated levels of circulating catecholamines and inflammatory mediators (Livingston et al., 2003; Robinson et al., 2006; Loftus et al., 2018). The early use of propranolol following severe traumatic head injury appears to be beneficial as it is known to blunt aspects of Cushing reflex which include early tachycardia, reduce hematopoietic progenitor cell mobilization, and results in a faster return to baseline of the inflammatory granulocyte colony-stimulating factor peak often seen with head injury (Fonseca et al., 2004; Cook et al., 2013; Bible et al., 2014) However, the degree to which propranolol benefits the mTBI patient is relatively unknown. Observational data suggests that early use of the drug, post-injury, likely controls hemodynamics and blood sugar with decreased catecholamine levels (Alali et al., 2017). Subsequently, the non-selective beta blocker, propranolol, has been shown to limit activation of these pathways in many injury paradigms; however, its role in bone marrow tissue function after rmTBI requires further investigation. Overall, our study suggests propranolol may impact rmTBI bone marrow tissue by altering metabolic pathways, and potentially limiting inflammation and epigenetic modifications.

Our data suggests that epigenetic changes occurring after rmTBI can be modulated by short term dosing of propranolol. Four genes important for epigenetic modifications that we further investigated based on significant expression changes across the three time points were *hdac6, camk1, vegfa, and usp7*. Collectively, these genes were all decreased one day after injury; yet, returned to sham saline levels 30 days later in the drug-treated bone marrow compared to non-treated bone marrow. *Vegfa*, *rock1*, *hmgb1*, *itgb1*, and *tfdp2* have roles in the differentiation ability of bone marrow stem cells transitioning from mesenchymal to epithelial phenotypes or the ability of epithelial determined stem cells differentiating back to mesenchymal phenotype. While these genes have important roles in other pathways such as apoptosis, oxidative stress, metabolism, autophagy, TGFβ, and intracellular signaling, the changes in the mRNA expression here reflect potential widespread bone marrow tissue epigenetic dysfunction impacting many pathways after mTBI.

One mRNA transcript that was impacting many of the possible signaling pathway changes was branched chain amino transferase 2 (*bcat2*). This enzyme appears to be important for mitochondrial protein and energy metabolism. Branch chain amino acids (BCAA; leucine, isoleucine, and valine) serve as precursors to many proteins and neurotransmitters (Holecek, 2018). The *bcat2* enzyme is pivotal for BCAA catabolism and helps in the generation of glutamic acid, glutamate, and glutamine (Dimou et al., 2022). BCAA levels have been noted to decrease following a TBI event and supplementation of BCAAs has been shown to improve post TBI cognitive recovery in humans (Aquilani et al., 2008; Sharma et al., 2018; Dickerman et al., 2022). The fact that we observed increased *bcat2* and mTOR pathway scores one day after injury but decreased levels 30 days later in TS bone marrow tissue may indicate that bcat2 expression in TP bone marrow could prove to have a restorative function. Bcat2, glutamate, and glutamate activity can also lead to PI-3 K/mTOR pathway activation. Integrin subunit beta 1 gene (ITGB1), produces a protein involved in PI-3K-AKT signaling. We found *itgb1* to be significantly decreased 7 and 30 days after TBI but that propranolol administration led to increased sham saline *itgb1* levels at these same timepoints. Accordingly, we found mTOR signaling at 30 days to be decreased in the drug-treated injury paradigm, while injury alone tissue displayed increases in this signaling pathway. Therefore, it is entirely possible that blocking beta receptors with propranolol helps reduce bone marrow metabolic processing of inflammatory mediators following traumatic injury.

Though understudied, rmTBIs appear to increase metabolism of amino acids, fatty acids, glucose, and glutamine one day after injury, but propranolol administration resulted in lower expression of genes in these pathways. Two genes involved in glucose metabolism and inflammation that we further investigated were sorbin and SH3 domain-containing protein 1 (*sorbs1*) and high mobility group box 1 (*hmgb1*). Little is known regarding *sorbs1* gene function apart from a role in insulin resistance (Chang et al., 2018). HMGB1 is a nuclear protein that can exhibit inflammatory behavior, as it can be released after injury to act on TLR4 or RAGE receptors depending on its oxidation state (Allette et al., 2014; Frank et al., 2015; Weber et al., 2015). Both genes display low levels in saline treated TBI bone marrow compared to propranolol treated TBI bone marrow. Importantly, TP bone marrow showed no changes from SS bone marrow 7 days after injury; however, TS bone marrow showed reductions in amino acid and glutamate metabolism. Interestingly, though both TS and TP bone marrow had decreased pathway scores 30 days after injury, propranolol treated rmTBI tissue displays substantially less expression, further displaying decreased bone marrow metabolism. This may result in a less inflamed bone marrow environment for stem cells and bone marrow derived immune cells to develop and mature. Collectively, TP bone marrow may respond more appropriately to future challenges.

The rmTBI paradigm also appears to disrupt signaling pathways for integrin signaling, JAK–STAT, oxidative stress, and TGFβ, as all these pathways were decreased with propranolol treatment when compared to untreated bone marrow tissue at both one day and 30 days after injury. In addition to the previously discussed changes in *itgb1*, *jak2* mRNA was increased in TP bone marrow 7 days after injury but restored back to sham levels 30 days after injury. TS bone marrow displayed substantially decreased *jak2* mRNA levels 30 days post injury. Activity in these pathways can lead to increased white blood cell modulation and inflammatory cytokine production (Kalra et al., 2022) For example, TGFβ production and activity lead to activation of immunosuppressive T cells, which may further increase inflammation by limiting injury resolution (Sanjabi et al., 2017; Simon et al., 2017). While we did not measure TGFβ protein, our data does support this premise, as we detected increased TGFβ signaling in saline treated TBI animals. Interestingly, TGFβ signaling was reduced in the TP group at 30 days. *Rock1*, *cul1*, and *tfdp2* are involved with different portions of the TGFβ signaling pathway. All were decreased 30 days after injury but treatment with propranolol after mTBI restored their mRNAs to sham saline levels 30 days after injury. These changes may be a compensation response as there was no difference in TGFβ signaling in propranolol treated bone marrow tissue at the one-week timepoint.

Overall, propranolol treatment accelerated the resolution of gene overexpression involved in cell cycle and delayed the expression of pro-inflammatory molecules (e.g., genes involved in osteoclast differentiation). Such differential kinetics could potentially provide a more favorable tissue recovery environment for TP by uncoupling cell proliferation and inflammation. This indicates that the therapeutic effects of propranolol treatment are largely timeline specific, and different molecules are operative along the timeline of TBI.

The observations herein are limited in that we did not measure which bone marrow cell types exhibited the mRNA transcript changes or possible changes in protein expression. Subsequently it is difficult to attribute a direct mechanism for drug action in the bone marrow tissue after rmTBI. However, several studies have documented that stress-induced, population-wide leukocyte shifts are associated with changes in the bone marrow. For example, stress-induced changes in innate immunity can reprogram neutrophils or monocytes and direct these cells to injury sites or back to the bone marrow. On the other hand, nervous system-mediated leukocyte shifts while reducing protect autoimmunity impairs immunity to disease challenge (Poller et al., 2022; Janssen et al., 2023).

Another uncontrolled variable was the systemic administration of propranolol. Direct injection into bone marrow tissue would allow for additional insight regarding direct or indirect effects of the drug on bone marrow tissue. Nevertheless, the fact that propranolol appears to produce long-term effects on the tissue suggests impactful changes on a number of signaling pathways including cellular metabolism, TGF beta signaling, integrin signaling, and JAK–STAT signaling pathways. An additional element that could not be controlled in this study was the use of a sham injury as a baseline gene expression control. The type of sham operation which is commonly utilized in mTBI rodent models objectively damages the rodent scalp. Evidence of changes in gene expression due to the traditional sham operation as a control has been shown to confer proinflammatory and morphological damage, which confounds some interpretation of conventional experimental brain injury models (Cole et al., 2011).

Further, studying the cellular effects of Propranolol would benefit from our recent work on Lithium (Li), which has broad implications from psychiatric disorders, dementia, and mTBI to head trauma (Maloney et al., 2019). Li is a medication long used to treat bipolar disorder and is currently under investigation for multiple nervous system disorders. Notably, Li’s modification of RNA levels depends on both RNA length and type (Maloney et al., 2019), and cotreatment studies of Li and propranolol are worth investigating. We also study transcriptomic, including non-coding RNAs, such as microRNAs (miRNA), from mechanistic to human brain studies (Chopra et al., 2021; Wang et al., 2022). We will test the effect of Propranolol on miRNA activity in the future. Finally, we plan to study Propranolol’s effect on epigenetics pathways and environmental factors (Lahiri et al., 2016; Maloney and Lahiri, 2016).

In summary, our study concludes that short term propranolol exposure causes differential gene expression changes impacting many bone marrow tissue pathways over the course of the post-injury month. Collectively, this could be an important step toward a better understanding of mechanisms which may be central to a better understanding of the short term and long-term events associated with mTBI within the peripheral compartment.

## Data availability statement

The raw data supporting the conclusions of this article will be made available by the authors, without undue reservation.

## Ethics statement

The animal study was reviewed and approved by Institutional Animal Care and Use Committee (IACUC) of the Indiana University School of Medicine.

## Author contributions

JS, DL, TH, AO, and FW conceived the project, designed the experiments, analyzed, discussed data, edited, and wrote the manuscript. TN designed experiments and analyzed data. JS and BD performed the experiments and analyzed the data. FW and TH responsible for funding acquisition. All authors contributed to the article and approved the submitted version.

## Funding

This research was funded in part from the Indiana State Department of Health’s Indiana Spinal Cord and Brain Injury Fund Research Grant Program (FW), Peer Reviewed Medical Research Program, Department of the Army W81XWH-18-1-0433 (FW), NIH/NINDS NS102415 (FW and AO), a pre-doctoral fellowship to JS and a post-doctoral fellowship to TN from Indiana Clinical and Translational Sciences Institute, NIHUL1TR002529 (S. Moe, PI). NIH/NIAID AI148282 (TH), VA Merit award BX002901 (TH). We also acknowledge support from NIA/NIH grants (P30AG072976, R56AG072810, and R21AG074539).

## Conflict of interest

The authors declare that the research was conducted in the absence of any commercial or financial relationships that could be construed as a potential conflict of interest.

## Publisher’s note

All claims expressed in this article are solely those of the authors and do not necessarily represent those of their affiliated organizations, or those of the publisher, the editors and the reviewers. Any product that may be evaluated in this article, or claim that may be made by its manufacturer, is not guaranteed or endorsed by the publisher.
